# Prognostic impact of postoperative systemic inflammatory response in patients with stage II/III gastric cancer

**DOI:** 10.1038/s41598-022-07098-3

**Published:** 2022-02-22

**Authors:** Kenji Kuroda, Takahiro Toyokawa, Yuichiro Miki, Mami Yoshii, Tatsuro Tamura, Hiroaki Tanaka, Shigeru Lee, Kazuya Muguruma, Masakazu Yashiro, Masaichi Ohira

**Affiliations:** Department of Gastroenterological Surgery, Osaka City University Graduate School of Medicine, 1-4-3 Asahimachi, Abeno-ku, Osaka, 545-8585 Japan

**Keywords:** Cancer, Gastrointestinal cancer

## Abstract

This study examined whether the systemic inflammatory response present in the early phase of the postoperative state correlates with long-term outcomes and to identify markers in patients with stage II/III gastric cancer. 444 consecutive patients who underwent radical gastrectomy for stage II/III gastric cancer were retrospectively reviewed. We evaluated maximum serum C-reactive protein (CRP_max_) and white blood cell count (WBC_max_), defined as the maximum serum CRP level and maximum WBC count during the interval from surgery until discharge, as systemic inflammation markers. In univariate analyses, CRP_max_, WBC_max_ and infectious complications were significantly associated with both overall survival (OS) (*p* < 0.001, *p* < 0.001 and *p* = 0.011, respectively) and relapse-free survival (RFS) (*p* < 0.001, *p* = 0.001 and *p* < 0.001, respectively). Multivariate analysis revealed that high-CRP_max_ (> 9.2 mg/dL) was an independent prognostic factor for OS (hazard ratio (HR) 1.68, 95% confidence interval (CI) 1.19–2.36, *p* = 0.003) and RFS (HR 1.56, 95% CI 1.12–2.18, *p* = 0.009), while WBC_max_ and infectious complications were not. CRP_max_, which reflects the magnitude of systemic inflammation induced by surgical stress and postoperative complications in the early phase after surgery, may be a promising prognostic indicator in patients with stage II/III gastric cancer who undergo curative resection.

## Introduction

Gastric cancer is the fifth most common fatality-causing and the third leading morbidity-causing carcinoma in the world^1^. Evolutions in diagnosis and multimodal therapy have improved treatment outcomes. However, the prognosis of gastric cancer patients with stage II or III disease remains insufficient. According to a retrospective analysis of nationwide data from the Japanese Gastric Cancer Association, 5-year overall survival rates for gastric cancer patients were 70.6% in stage II, 53.6% in stage IIIA and 34.8% in stage IIIB^2^. The ACTS-GC trial showed that 5-year overall survival rates of patients who underwent radical gastrectomy with D2 lymph node dissection followed by adjuvant chemotherapy were 84.2% in stage II, 67.1% in stage IIIA and 50.2% in stage IIIB. To further improve the prognosis of patients treated with standard treatment, identifying prognostic factors is important.

Inflammation has been revealed to be closely related to the progression and metastasis of cancer cells^3^. Several studies have shown that postoperative infectious complications correlate with postoperative recurrence and poor prognosis in various malignancies, including gastric cancer^4–7^. Systemic inflammatory responses caused by infectious complications could induce not only the proliferation of residual cancer cells, but also declines in host immunity, which may lead to worsened prognosis^8,9^. On the other hand, in the postoperative early phase, surgical stress also induces systemic inflammatory responses. Several recent studies have focused on the association between long-term outcome and postoperative systemic inflammatory response caused by surgical stress and complications in gastroenterological cancer^10–13^. However, the significance of postoperative inflammatory response in the early phase after surgery and its markers in patients with gastric cancer remain unclear. In addition, appropriate markers of systemic inflammation that correlate with prognosis remain uncertain.

The aim of this study was to examine whether the systemic inflammatory response caused in the early phase of the postoperative state correlates with prognosis in advanced gastric cancer patients who undergo curative resection. We evaluated C-reactive protein (CRP) and white blood cell (WBC) count as simple and versatile markers of postoperative systemic inflammation in clinical practice that may be suitable for detecting the magnitude of inflammatory reaction.

## Results

### Predictive ability and cut-off values for CRP_max_ and WBC_max_

Median CRP_max_ and WBC_max_ were 10.4 mg/dL (interquartile range [IQR], 7.3–14.9 mg/dL) and 11,000/mm^3^ (IQR, 9,200–13,900/mm^3^), respectively. Areas under the curve predicting 5-year OS were 0.615 for CRP_max_ and 0.573 for WBC_max_, respectively. Values of 9.2 mg/dL for CRP_max_ and 15,100/mm^3^ for WBC_max_ provided maximal Youden indices, and were thus set as the cut-off values. We classified 258 patients (58.1%) and 186 patients (41.9%) as having high-CRP_max_ and low-CRP_max_, respectively, and 83 patients (18.7%) and 361 patients (81.3%) as having high-WBC_max_ and low-WBC_max_, respectively.

### Correlations between CRP_max_, WBC_max_ and clinicopathological variables

Table 1 shows the associations between CRP_max_, WBC_max_ and clinicopathological variables. CRP_max_ was significantly associated with operative procedure, operation time, blood loss, tumor size, tumor location, and postoperative infectious complications (*p* < 0.001 each). WBC_max_ was significantly associated with operative procedure, operation time, blood loss, tumor size, tumor location, and postoperative infectious complications (*p* < 0.001 each).

**Table 1:** Patient characteristics.

Characteristics	High-CRP_max_ (n = 258)	Low-CRP_max_ (n = 186)	*p* value	High-WBC_max_ (n = 83)	Low-WBC_max_ (n = 361)	*p* value
Age (years)			0.909			0.775
< 75	199 (77.1%)	142(76.3%)		65 (78.3%)	276 (76.5%)	
≥ 75	59 (22.9%)	44 (23.7%)		18 (21.7%)	85 (23.5%)	
Sex			0.136			0.336
Male	195 (75.6%)	109 (58.6%)		61 (67.3%)	243 (73.5%)	
Female	63 (24.4%)	77 (41.4%)		22 (32.7%)	118 (26.5%)	
BMI (kg/m^2^)			0.141			0.852
< 18.5	26 (10.1%)	28 (15.1%)		9 (10.8%)	45 (12.5%)	
≥ 18.5	232 (89.9%)	158 (84.9%)		74 (89.2%)	316 (87.5%)	
PS			0.658			0.566
0	201 (77.9%)	149 (80.1%)		63 (75.9%)	287 (79.5%)	
1–2	57 (22.1%)	37 (19.9%)		20 (24.1%)	74 (20.5%)	
Operative approach			0.113			0.326
Open	246 (95.3%)	170 (91.4%)		80 (96.4%)	236 (93.1%)	
Laparoscopic	12 (4.7%)	16 (8.6%)		3 (3.6%)	25 (6.9%)	
Operative procedure			< 0.001			< 0.001
DG, PG	122 (46.9%)	140 (75.3%)		24 (28.9%)	238 (65.7%)	
TG	136 (52.7%)	46 (24.7%)		59 (71.1%)	123 (34.1%)	
Operation time (min)	230 [199–282]	194 [171–233]	< 0.001	250 [204–293]	210 [180–248]	< 0.001
Blood loss (ml)	448 [290–660]	253 [150–394]	< 0.001	530 [310–778]	330 [190–500]	< 0.001
Tumor size (mm)	55 [15–200]	45 [10–130]	< 0.001	64 [15–90]	50 [10–200]	< 0.001
Depth of invasion			0.221			0.248
T1–2	45 (17.4%)	42 (22.6%)		12 (14.5%)	75 (20.8%)	
T3–4	213 (82.6%)	144 (77.4%)		71 (85.5%)	286 (79.2%)	
Lymph node metastasis			0.285			0.89
N0	66 (25.6%)	57 (30.6%)		24 (28.9%)	99 (27.4%)	
N1–3	192 (74.4%)	129 (69.4%)		59 (71.1%)	262 (72.6%)	
pStage^†^			0.106			0.229
II	123 (47.7%)	104 (55.9%)		37 (44.6%)	190 (52.6%)	
III	135 (52.3%)	82 (44.1%)		46 (55.4%)	171 (47.4%)	
Tumor location			< 0.001			< 0.001
U	98 (38.0%)	31 (16.7%)		38 (45.8%)	91 (25.2%)	
M	68 (26.4%)	80 (43.0%)		20 (24.1%)	128 (35.5%)	
L	79 (30.6%)	71 (38.2%)		17 (20.5%)	133 (36.8%)	
UML	13 (5.0%)	4 (2.2%)		8 (9.6%)	9 (2.5%)	
Histology			0.408			0.135
Differentiated	121 (46.9%)	79 (42.5%)		44 (53.0%)	156 (43.2%)	
Undifferentiated	137 (53.1%)	107 (58.5%)		39 (47.0%)	205 (56.8%)	
Postoperative infectious complication*			< 0.001			< 0.001
Absent	190 (73.6%)	183 (98.4%)		45 (54.2%)	328 (90.9%)	
Present	68 (26.4%)	3 (1.6%)		38 (45.8%)	33 (9.1%)	
Adjuvant chemotherapy			0.526			0.804
Absent	60 (23.3%)	49 (26.3%)		19 (22.9%)	90 (26.3%)	
Present	198 (76.7%)	137 (73.7%)		64 (77.1%)	271 (73.7%)	

Values in parentheses are percentages unless indicated otherwise; values are all median (i.q.r.)

*BMI* body mass index, *PS* performance status, *DG* distal gastrectomy, *PG* proximal gastrectomy, *TG* total gastrectomy, *U* upper, *M* middle, *L* lower.

^†^According to the 7th edition of the International Union Against Cancer.

*Intra-abdominal abscess, anastomotic leakage, pancreatic fistula, pneumonia, surgical site infection, acute cholecystitis, and enteritis Grade ≥ 2 based on the Clavien-Dindo classification.

### Survival and prognostic factors

Median follow-up for survivors was 88 months (IQR, 73–122 months). Thirteen patients were lost to follow-up within 5 years, with 16 months as the shortest follow-up period for survivors. Recurrence was observed in 162 cases, and median duration to recurrence was 15 months (IQR, 8–29 months). A total of 200 deaths were observed.

**Figure 1:**
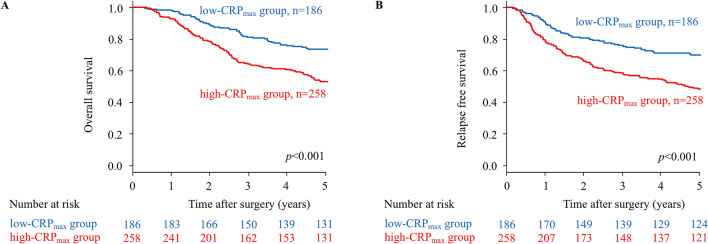
Kaplan–Meier survival curves of overall survival (OS) and relapse-free survival (RFS) according to CRP_max_ in patients with stage II/III gastric cancer. (**A**) The five-year OS rates are 66.1% in the low-CRP_max_ group and 45.6% in the high-CRP_max_ group (*p* < 0.001). (**B**) The five-year RFS rates are 63.5% in the low-CRP_max_ group and 44.4% in the high-CRP_max_ group (*p* < 0.001).

**Figure 2:**
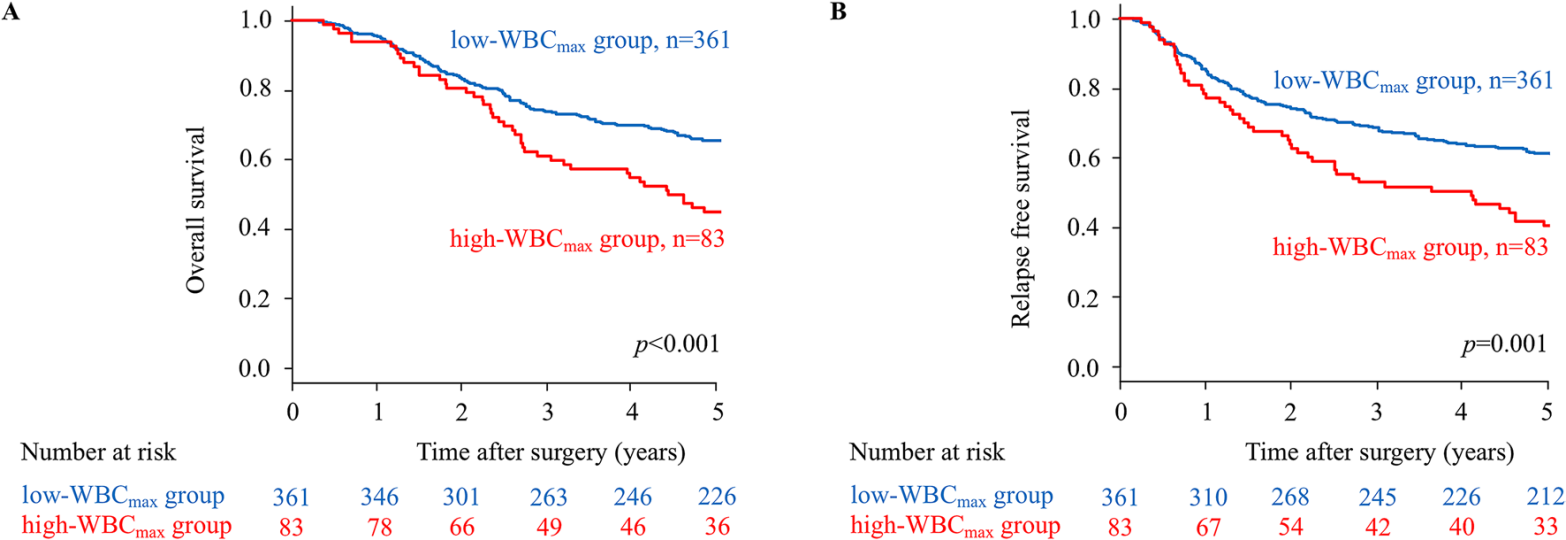
Kaplan–Meier survival curves of overall survival (OS) and relapse-free survival (RFS) according to WBC_max_ in patients with stage II/III gastric cancer. (**A**) The five-year OS rates were 58.1% in the low-WBC_max_ group and 37.4% in the high-WBC_max_ group (*p* < 0.001). (**B**) The five-year RFS rate were 56.1% in the low-WBC_max_ group and 36.1% in the high-WBC_max_ group (*p* = 0.001).

Five-year OS and RFS rates for the entire study population were 59.0% and 55.2%, respectively. OS and RFS rates in patients with high-CRP_max_ were significantly poorer than those of patients with low-CRP_max_ (*p* < 0.001 each) (Fig. 1). OS and RFS rates in patients with high-WBC_max_ were also significantly poorer than those of patients with low-WBC_max_ (*p* < 0.001 and *p* = 0.001, respectively) (Fig. 2).

Results of uni- and multivariate analyses for OS and RFS are summarized in Tables 2 and 3, respectively. Univariate analyses for OS revealed significant correlations with CRP_max_, WBC_max_, age, BMI, PS, operative procedure, operative approach, operation time, blood loss, pathological stage, postoperative infectious complications, and adjuvant chemotherapy. Multivariate analysis for OS indicated that CRP_max_ (HR 1.68 95%CI 1.19–2.36, *p* = 0.003), BMI (HR 0.59, 95%CI 0.40–0.86, *p* = 0.007), PS (HR 1.43, 95%CI 1.01–2.04, *p* = 0.047), pathological stage (HR 2.41, 95%CI 1.78–3.26, *p* < 0.001) and adjuvant chemotherapy (HR 0.68, 95%CI 0.48–0.95, *p* = 0.007) were independent prognostic factors. Univariate analyses for RFS revealed significant correlations with CRP_max_, WBC_max_, age, BMI, PS, operative procedure, operative approach, blood loss, pathological stage, and postoperative infectious complications. Multivariate analysis for RFS indicated CRP_max_ (HR 1.56, 95%CI 1.12–2.18, *p* = 0.009), age (HR 1.42, 95%CI 1.01–1.98, *p* = 0.042), BMI (HR 0.66, 95%CI 0.45–0.96, *p* = 0.032), PS (HR 1.30, 95%CI 1.04–1.61, *p* = 0.020) and pathological stage (HR 2.23, 95%CI 1.67–2.98, *p* < 0.001) as independent prognostic factors.

**Table 2:** Univariate and multivariate analysis for overall survival.

Variable	Univariate analysis		Multivariate analysis	
	Hazard ratio (95% CI)	*p* value	Hazard ratio (95% CI)	*p* value
CRP_max_ > 9.2 mg/dL	1.93 (1.43–2.61)	< 0.001	1.68 (1.19–2.36)	0.003
WBC_max_ > 15,100 /μL	1.72 (1.25–2.37)	< 0.001	0.99 (0.68–1.44)	0.972
Age ≥ 75 years	1.86 (1.37–2.52)	0.002	1.37 (0.95–1.97)	0.093
Female (vs. Male)	0.91 (0.67–1.23)	0.519		
BMI ≥ 18.5 kg/m^2^	0.64 (0.44–0.93)	0.021	0.59 (0.40–0.86)	0.007
PS > 0	1.81 (1.33–2.47)	< 0.001	1.43 (1.01–2.04)	0.047
Operation time	1.002 (1.001–1.004)	< 0.001	1.001 (0.99–1.003)	0.487
Blood loss	1.001 (1.000–1.001)	0.011	1.000 (0.99–1.000)	0.898
Operative procedure: TG	1.88 (1.43–2.46)	< 0.001	1.33 (0.96–1.85)	0.086
Operative approach: laparoscopy	0.45 (0.21–0.96)	0.039	0.62 (0.28–1.38)	0.241
Histology: undifferentiated	1.20 (0.90–1.59)	0.207		
Stage III (vs. II)	2.50 (1.87–3.35)	< 0.001	2.41 (1.78–3.26)	< 0.001
Postoperative infectious complication	1.64 (1.12–2.41)	0.011	1.18 (0.80–1.73)	0.410
Adjuvant chemotherapy	0.71 (0.52–0.97)	0.029	0.68 (0.48–0.95)	0.007

**Table 3:** Univariate and multivariate analysis for relapse free survival.

Variable	Univariate analysis		Multivariate analysis	
	Hazard ratio (95% CI)	*p* value	Hazard ratio (95% CI)	*p* value
CRP_max_ > 9.2 mg/dL	1.83 (1.37–2.44)	< 0.001	1.56 (1.12–2.18)	0.009
WBC_max_ > 15,100 /μL	1.67 (1.22–2.29)	0.001	1.02 (0.71–1.48)	0.907
Age ≥ 75 years	1.74 (1.29–2.34)	< 0.001	1.42 (1.01–1.98)	0.042
Female (vs. Male)	0.89 (0.66–1.20)	0.441		
BMI ≥ 18.5 kg/m^2^	0.71 (0.48–1.03)	0.070	0.66 (0.45–0.96)	0.032
PS > 0	1.81 (1.34–2.45)	< 0.001	1.30 (1.04–1.61)	0.020
Operation time	1.002 (1.00–1.004)	0.051	1.000 (1.00–1.003)	0.838
Blood loss	1.001 (1.00–1.001)	0.002	1.000 (0.99–1.000)	0.813
Operative procedure: TG	1.75 (1.35–2.28)	< 0.001	1.24 (0.90–1.71)	0.183
Operative approach: laparoscopy	0.43 (0.20–0.92)	0.030	0.61 (0.28–1.35)	0.222
Histology: undifferentiated	1.15 (0.87–1.51)	0.326		
Stage III (vs. II)	2.57 (1.93–3.42)	< 0.001	2.23 (1.67–2.98)	< 0.001
Postoperative infectious complication	1.79 (1.28–2.51)	< 0.001	1.23 (0.84–1.80)	0.279
Adjuvant chemotherapy	0.78 (0.58–1.06)	0.115		

### Subgroup analysis for OS according to adjuvant chemotherapy and postoperative infectious complications

**Figure 3:**
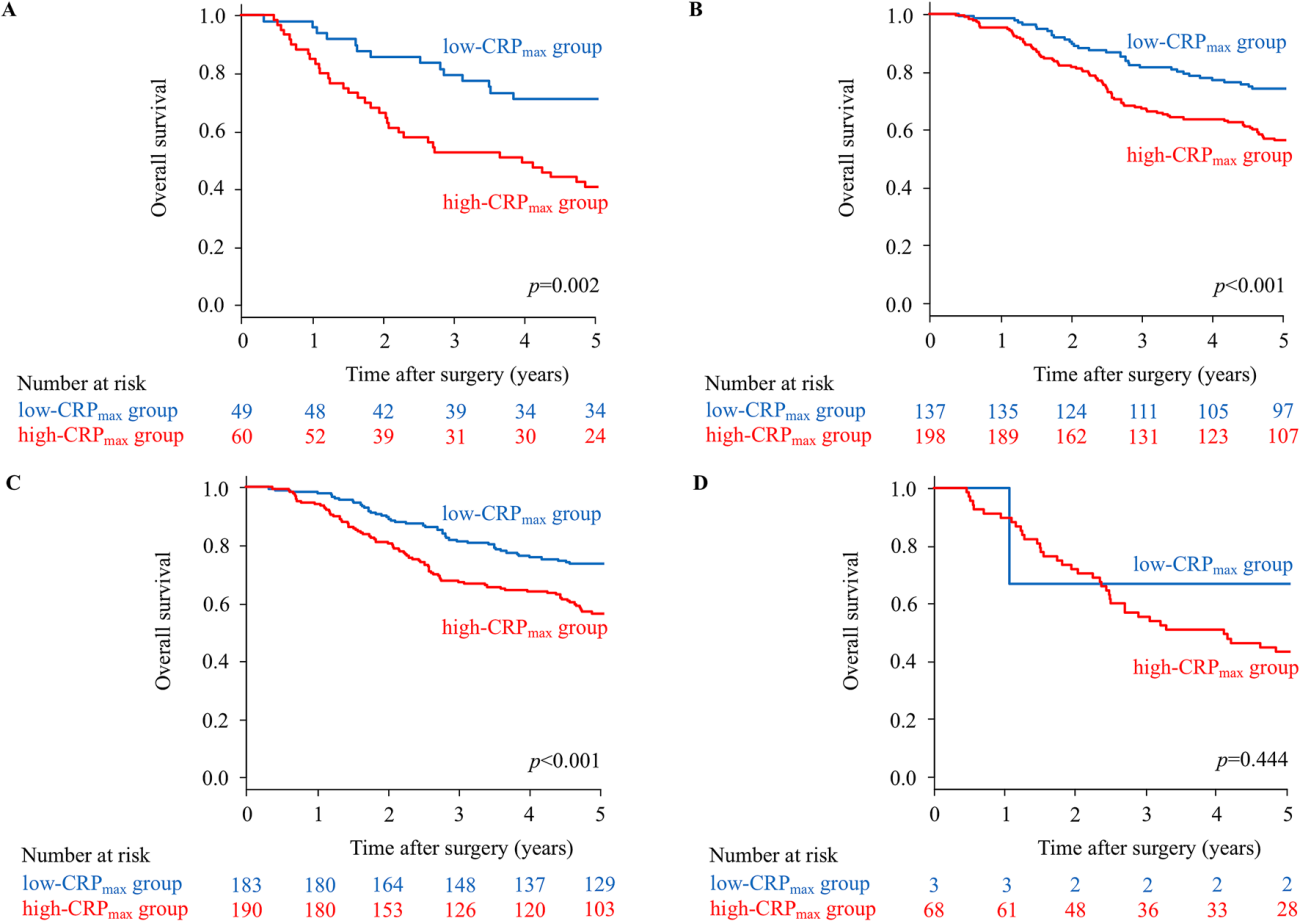
Kaplan–Meier survival curves of overall survival (OS) according to CRP_max_ in patients without adjuvant chemotherapy (**A**, *p* = 0.002), in patients with adjuvant chemotherapy (**B**, *p* < 0.001), in patients without postoperative infectious complications (**C**, *p* < 0.001), in patients with postoperative infectious complications (**D**, *p* = 0.444).

**Figure 4:**
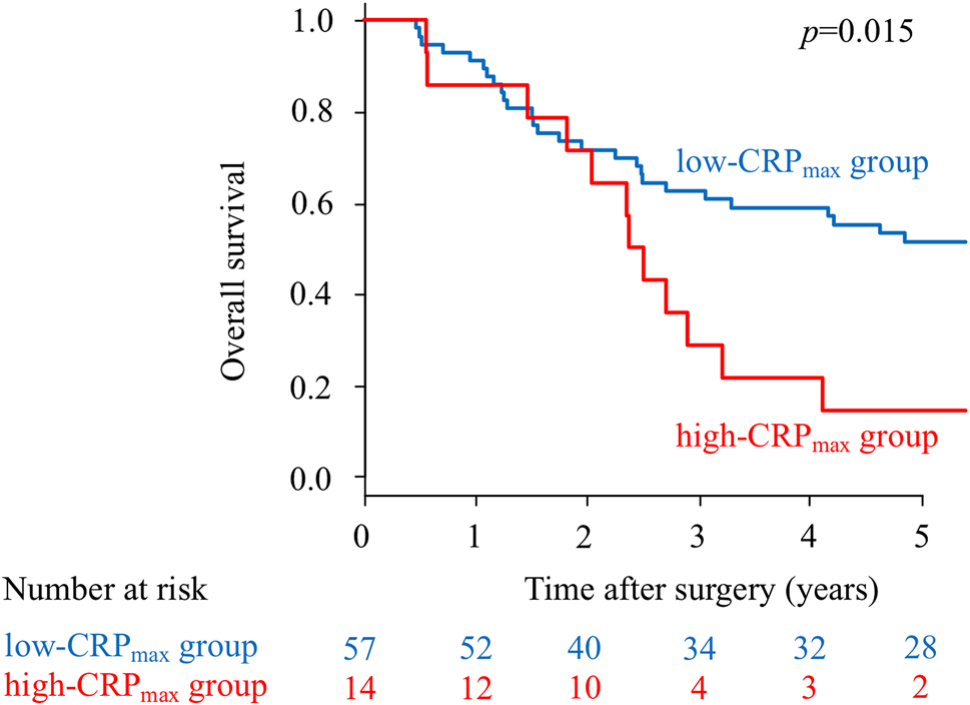
Kaplan–Meier survival curves of overall survival (OS) according to CRP_max_ in patients with postoperative infectious complications (*p* = 0.015).

Figure 3A and B show the Kaplan–Meier survival curves comparing OS for CRP_max_ according to adjuvant chemotherapy. In patients with and without adjuvant chemotherapy, OS rates were significantly lower in the high-CRP_max_ group than in the low-CRP_max_ group (*p* = 0.002 and *p* < 0.001, respectively). Figure 3C and D show the Kaplan–Meier survival curves comparing OS for CRP_max_ according to postoperative infectious complications. In patients without postoperative infectious complications, OS rates were significantly lower in the high-CRP_max_ group than in the low-CRP_max_ group (*p* < 0.001), whereas in the presence of postoperative infectious complications, no significant difference was observed between groups (*p* = 0.444); this was attributed to the fact that only 3 patients were included in the low-CRP_max_ group. Therefore, we used another cut-off value of 26.2 mg/dL for patients with postoperative infectious complications, determined based on the same method used for the entire cohort. In patients with postoperative infectious complications, OS rates were significantly lower in this new high-CRP_max_ (> 26.2 mg/dL) group than in the new low-CRP_max_ (≤ 26.2 mg/dL) group (*p* = 0.015) (Fig. 4).

**Figure 5:**
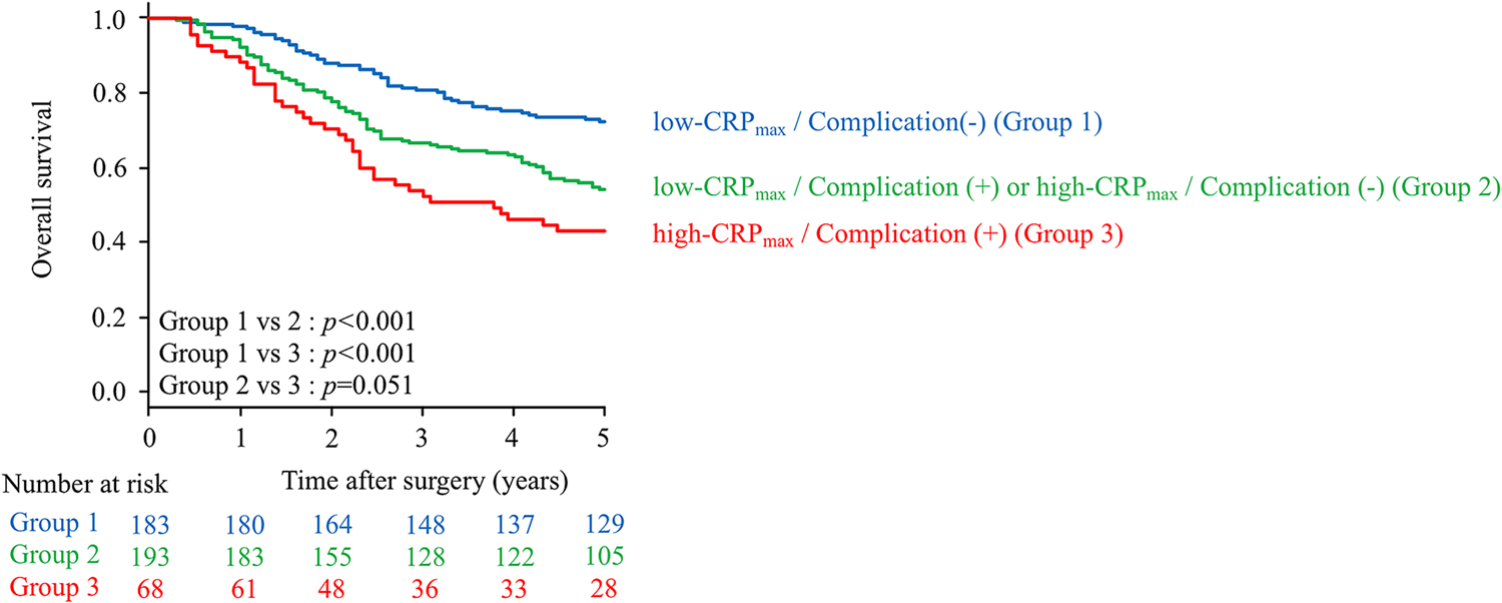
Kaplan–Meier survival curves of overall survival (OS) according to CRP_max_ and postoperative infectious complication status (Group 1, high-CRP_max_ with postoperative infectious complications; Group 2, either high-CRP_max_ or the presence of postoperative infectious complications; Group 3, low-CRP_max_ without postoperative infectious complications). The five-year OS rates in Groups 1, 2 and 3 were 73.5%, 56.5% and 43.1%, respectively (Group 1 vs Group 2: *p* < 0.001, Group 1 vs Group 3: *p* < 0.001, Group 2 vs Group 3: *p* = 0.051).

We classified patients according to CRP_max_ and postoperative infectious complication status into three groups as follows: Group 1, high-CRP_max_ with postoperative infectious complications; Group 2, either high-CRP_max_ or the presence of postoperative infectious complications; and Group 3, low-CRP_max_ without postoperative infectious complications. As shown in Fig. 5, this classification allowed clear separation of survival curves. The five-year OS rates in Groups 1, 2 and 3 were 73.5%, 56.5% and 43.1%, respectively (Group 1 vs Group 2: *p* < 0.001, Group 1 vs Group 3: *p* < 0.001, Group 2 vs Group 3: *p* = 0.051).

### Correlation between postoperative inflammatory response and site of recurrence

Sites of first recurrence according to CRPmax and WBCmax are shown in Table 4. The proportion of patients with hematogenous recurrence was significantly higher in the high-CRPmax group (16.3%) and the high-WBCmax group (20.5%) than in the low-CRPmax group (3.8%, *p* < 0.001) and the low-WBCmax group (8.9%), respectively. Whereas no significant difference was evident between CRPmax and peritoneal recurrence, lymph node recurrence and local recurrence, and the same applied to WBC_max_.

**Table 4:** Recurrence patterns.

Variable	High-CRP_max_ (n = 258)	Low-CRP_max_ (n = 186)	*p* value
Peritoneum	43 (16.7%)	24 (12.9%)	0.347
Lymph nodes	36 (14.0%)	22 (11.8%)	0.571
Hematogenous	43 (16.7%)	7 (3.8%)	< 0.001
-Liver	26 (10.1%)	5 (2.7%)	0.002
-Lung	3 (1.2%)	0 (0.0%)	0.268
-Bone	8 (3.1%)	1 (0.5%)	0.086
-Brain	1 (0.4%)	0 (0.0%)	1.000
-Others	5 (1.9%)	1 (0.5%)	0.408
Local	1 (0.4%)	2 (1.1%)	0.574
Total	123 (47.7%)	55 (29.6%)	< 0.001

## Discussion

The present study investigated correlations between postoperative systemic inflammatory response and prognosis in advanced gastric cancer patients who underwent curative resection, using the maximum postoperative CRP level and WBC count during hospitalization after gastrectomy as markers of systemic inflammation. We found that CRP_max_ with a cut-off of 9.2 mg/dL was an independent prognostic factor, but WBC_max_ was not. Further, CRP_max_ affected OS and RFS independent of postoperative infectious complications. These results suggest that systemic inflammatory response as represented by CRP_max_ in the early phase after surgery induced by surgical stress and complications is associated with recurrence and survival in patients with stage II/III gastric cancer.

This study revealed CRP_max_ as an independent prognostic indicator for OS and RFS in patients with stage II/III gastric cancer who underwent curative resection. Consistent with our results, previous studies have demonstrated that postoperative systemic inflammatory response correlated with recurrence and poor prognosis in gastroenterological cancers including gastric cancer^11,12,14^, esophageal cancer^15,16^, and colorectal cancer^17^. However, the optimal marker remains uncertain. Because the magnitude of surgical stress and postoperative complications can differ markedly among cancers, optimal markers and the respective cut-off values should be determined for each cancer. Okumura et al. revealed postoperative prolonged hyperthermia, defined as a maximum body temperature > 38 °C for ≥ 4 days, as an independent prognostic factor for OS and RFS in patients with stage II/III gastric cancer^11^. However, whether postoperative prolonged hyperthermia affected survival independent of the occurrence of postoperative infectious complications was unclear because postoperative complications were strongly associated with prolonged hyperthermia in that study. We evaluated WBC_max_ and CRP_max_ as markers that are not only commonly used to estimate the magnitude of inflammation in postoperative management, but also simple and readily available in daily clinical practice. Consequently, we found that CRP_max_ was an independent prognostic factor for OS and RFS, but WBC_max_ was not. Similarly, Saito et al.^12^ demonstrated that postoperative CRP_max_ (≥ 12 mg/dL) was an independent prognostic factor for RFS in advanced gastric cancer, although postoperative WBC_max_ was not. Further, this finding was validated in their subsequent large-scale multicenter study^14^. Postoperative CRP_max_ could provide a useful marker to predict prognosis in patients with stage II/III gastric cancer who undergo curative resection.

Interestingly, the high-CRP_max_ group in the present study was significantly associated with hematogenous metastasis as the pattern of recurrence. Similar to our result, Kurokawa et al. reported that liver metastasis, but not peritoneal or lymph node metastasis, was significantly more frequent in the high-CRP_max_ (≥ 12 mg/dL) group than in the low-CRP_max_ group. Although the exact mechanisms underlying this association between higher postoperative inflammation and hematogenous metastasis remain unclear, some potential explanations can be considered. First, host immunosuppressive influences such as impairment of cellular immunity caused by the surgical stress could negatively impact on circulating tumor cells (CTCs) and micro-metastatic cancer cells. Although natural killer cells and macrophages play important roles in eliminating CTCs and preventing the formation of metastases, both the cytotoxicity of natural killer cells and macrophage function are reportedly impaired in proportion to the extent and magnitude of surgery^18,19^. Extraperitoneal tumor growth was demonstrated to be accelerated accompanied by suppressed natural killer cell cytotoxicity after surgery^20^. Second, systemic inflammation could accelerate the adhesion of CTCs to distant organs. E-selectin up-regulation, induced by inflammatory cells and pro-inflammatory cytokines such as interleukin (IL)-1 and tumor necrosis factor (TNF)-α, has been shown to promote recruitment of CTCs to the vascular endothelium^21–23^. Third, growth factors and cytokines such as TNF-α, vascular endothelial growth factor and IL-6 induced by the inflammatory response could promote the proliferation and metastasis of residual cancer cells^24^. Thus, not only optimizing surgical procedures, but also immunomodulatory approaches and anti-inflammatory approaches in the perioperative period might improve oncological outcomes.

In the present study, postoperative infectious complications were not an independent prognostic factor for OS and RFS, despite significant associations with those outcomes in univariate analyses. Some studies have demonstrated that postoperative complications correlate with prognosis in gastric cancer patients^4,5^, whereas others have not^25,26^. This inconsistency may be attributable to differences in the definition and frequency of postoperative complications among studies. Moreover, most studies did not enter postoperative inflammatory responses (including surgical stress) into multivariate analyses for survival. Our findings suggest that systemic inflammatory response induced by not only postoperative complications, but also surgical stress is more important than the actual postoperative complications as a prognostic factor in patients with advanced gastric cancer.

In the present study, because the 71 patients with postoperative infectious complications included only three patients with high-CRP_max_, we evaluated another cut-off value of CRP_max_ for patients with postoperative infectious complications. Consequently, patients with postoperative infectious complications and a high-CRP_max_ of > 26.2 mg/dL revealed poorer OS than those with low-CRP_max_≤ 26.2 mg/dL. This finding suggests that the magnitude of systemic inflammatory response caused by infection could affect OS. When postoperative infectious complications occur, early diagnosis and appropriate treatment for infectious complications may be important to reduce systemic inflammatory response and improve long-term outcomes in patients with stage II/III gastric cancer.

From Japan, some important surgical randomized controlled trials for advanced gastric cancer have been reported by the Japan Clinical Oncology Group (JCOG). The JCOG9501 study compared D2 lymphadenectomy alone with D2 lymphadenectomy plus para-aortic nodal dissection for advanced gastric cancer without clinical para-aortic lymph node metastasis^27^. The JCOG0110 study compared spleen preservation with splenectomy for advanced proximal gastric cancer not involving the greater curvature^28^. The JCOG1001 study compared non-bursectomy with bursectomy for advanced gastric cancer with cT3 (SS)-cT4b (SI)^29^. However, none of these studies demonstrated the prognostic efficacy of extended surgery. One reason why extended surgery uniformly failed to improve survival may be that the negative impact on residual cancer cells of the systemic inflammatory response involved in surgical stress and postoperative complications may offset any positive impact of extended surgery. Furthermore, the JCOG9502 study compared an abdominal-transhiatal approach with a left thoracoabdominal approach for advanced gastric cancer with esophageal invasion of ≤ 3 cm revealed worse survival in the left thoracoabdominal approach group^30^. In the JCOG9502 study, the rate of postoperative complications was higher in the left thoracoabdominal approach group and surgical stress was obviously larger in that same group. The impact of postoperative systemic inflammatory response induced by surgical stress and postoperative complications on survival may warrant more attention from general surgeons.

This study has some potential limitations that should be considered when interpreting the results. First, this retrospective study was conducted at a single institution and sample size was relatively small. Second, the present study showed heterogeneity in the adjuvant chemotherapy regimens, because the indications for and standard regimens of adjuvant chemotherapy had not been established until 2007, when the results of the ACTS-GC trial confirmed the efficacy of S-1 as adjuvant chemotherapy for stage II/III gastric cancer^31^. However, in the subgroup analysis with or without adjuvant chemotherapy, OS rates were significantly lower in the high-CRP_max_ group than in the low-CRP_max_ group. Prospective large-scale validation studies are needed to confirm our findings.

## Conclusion

CRP_max_, which reflects the magnitude of systemic inflammation induced by surgical stress and postoperative complications in the early phase after surgery, was associated with oncologic outcome in patients with stage II/III gastric cancer who underwent curative resection. Our findings suggest that surgeons should not underestimate the prognostic impact of surgical stress and postoperative complications in the management of advanced gastric cancer. To improve long-term outcomes for advanced gastric cancer patients, reducing surgical stress and postoperative complications may be important.

## Methods

### Patients

This retrospective analysis investigated consecutive patients who underwent radical gastrectomy for gastric cancer at Osaka City University Hospital (Osaka, Japan) between January 2000 and December 2013. Patients diagnosed with stage II/III gastric cancer on postoperative pathological examination were enrolled in this study. We excluded 23 patients who underwent neoadjuvant chemotherapy, 33 patients with concomitant multiple cancers, 27 patients with R1/2 resection, 9 patients with specific histological type, 6 patients with perioperative death, and 13 patients for whom the full set of clinical data was not available. Ultimately, 444 patients were included in this study. The Osaka City University Ethics Committee approved this retrospective study of clinical data (approval no. 4386), which was conducted in accordance with the principles of the Declaration of Helsinki.

### Data collection and evaluation of postoperative inflammatory response

We evaluated clinicopathological characteristics including age, sex, body mass index (BMI), Eastern Cooperative Oncology Group performance status (PS), blood test examination data, operative approach and procedure, operation time, blood loss, tumor size, depth of invasion, lymph node metastasis, pathological stage, tumor location, histology, macroscopic type, lymphatic invasion, venous invasion, postoperative infectious complication, and adjuvant chemotherapy from medical records. Tumors were staged according to the third English edition of the Japanese classification of gastric carcinoma^32^. Subjects were categorized according to age as elderly (≥ 75 years) and non-elderly (< 75 years), and according to BMI as underweight (BMI < 18.5 kg/m^2^) and normal or overweight (BMI ≥ 18.5 kg/m^2^). In the present study, postoperative infectious complications included intra-abdominal abscess, anastomotic leakage, pancreatic fistula, pneumonia, surgical site infection, acute cholecystitis, and enteritis of Grade 2 or higher according to the Clavien-Dindo classification^33^.

Serum CRP and WBC count were measured routinely on postoperative day (POD) 1, POD 3, and POD 6 or 7. In addition, serum CRP and WBC count were assessed when postoperative complications were suspected and when treatment efficacy was evaluated. We defined the maximum serum CRP level (CRP_max_) and maximum WBC count (WBC_max_) as the highest levels of those parameters identified during the entire interval from surgery until discharge. To set cut-off values for CRP_max_ and WBC_max_, time-dependent receiver operating characteristic (ROC) curve analyses for 5-year overall survival (OS) as the endpoint were calculated, and maximal Youden indices were estimated. All patients were classified as having high or low values according to these cut-offs. In the present study, postoperative infectious complications were defined as postoperative complications accompanied by elevated CRP needing antibiotics, drainage and surgery. To diagnose infectious complications, blood examination, measurement of amylase in drain fluid, and imaging studies, such as computed tomography, ultrasonography and contrast swallow, were performed based on clinical suspicion. The attending surgeons performed each examination and recorded the results. Consequently, we included 24 cases of anastomotic leakage, 24 cases of pancreatic fistula, 7 cases of intra-abdominal abscess, 7 cases of surgical site infection, 3 cases of pneumonia, 2 cases of acute cholecystitis, 1 case of acute pancreatitis, 1 case of enteritis, 1 case of urinary tract infection, and 1 case of catheter-related blood stream infection as Grade 2 or higher according to the Clavien-Dindo classification^15^.

### Treatment and follow-up

Surgical procedures were determined according to tumor size, location, and the status of resection margins. Laparoscopic surgery was preferred when clinical stage was less than stage IB. In principle, adjuvant chemotherapy with oral fluoropyrimidines (5-fluorouracil, uracil-tegafur, doxifluridine, or S-1) was administered after obtaining written informed consent, except for patients with pathological T1. Patients were routinely followed-up every 4 months for the first 2 years, every 6 months for the next 3 years, and annually thereafter. Each follow-up examination included physical examinations, routine blood tests, measurements of tumor marker levels, and contrast-enhanced computed tomography of the abdomen. These same examinations were also performed when recurrence was suspected. Recurrence was diagnosed according to the findings from these examinations. We contacted patients, family members, or their referring physicians to obtain appropriate follow-up data if the patient had not presented for follow-up.

### Statistical analysis

Statistical analysis was performed using R for Mac OS X version 3. 5. 2 (Saitama Medical Center, Jichi Medical University, Saitama, Japan), which is a graphical user interface for R (The R Foundation for Statistical Computing, Vienna, Austria). The χ^2^ test or Fischer’s test was used to analyze associations between categorical variables, and the Mann–Whitney U test was used to compare continuous variables. Overall survival (OS) and relapse-free survival (RFS) were defined as the time from the date of surgery to the date of last follow-up or death, and the time from the date of surgery to the date of confirmed recurrence or death, respectively. OS and RFS were calculated using Kaplan–Meier methods, and survival curves were compared by log-rank testing. Uni- and multivariate analyses for OS and RFS were conducted with Cox proportional hazards models. We performed multivariate analyses including variables with values of *p* < 0.1 in univariate analysis. Hazard ratios (HRs) and 95% confidence intervals (CIs) were calculated. We considered values of *p* < 0.05 as significant.

## Abbreviations

CRP: C-reactive protein
WBC: White blood cell
BMI: Body mass index
PS: Performance status
DG: Distal gastrectomy
PG: Proximal gastrectomy
TG: Total gastrectomy
U: Upper
M: Middle
L: Lower
OS: Overall survival
RFS: Relapse-free survival
